# Lactate as a Master Regulator of Immune Suppression: From Metabolic Waste to Epigenetic Checkpoint in Colorectal Cancer

**DOI:** 10.3390/ijms27167495

**Published:** 2026-08-21

**Authors:** Beiyan Chen, Shuang Gao, Xin Chen, Qingping Shi, Mingli Shen, Jieru Han

**Affiliations:** 1 School of Basic Medical Sciences, Heilongjiang University of Chinese Medicine, Harbin 150040, China; chenbeiyanabc@163.com (B.C.); gaoshuangacc@163.com (S.G.); sqp17326705862@163.com (Q.S.); 18309474893@163.com (M.S.); 2 First Clinical Medical College, Heilongjiang University of Chinese Medicine, Harbin 150040, China; radinen@outlook.com

**Keywords:** colorectal cancer, lactate, histone lactylation, tumor microenvironment, metabolic checkpoint, immunotherapy resistance

## Abstract

Colorectal cancer, especially the microsatellite-stable subtype, which accounts for 85% to 95% of cases, resists immune checkpoint inhibitors largely due to metabolic reprogramming in the tumor microenvironment. Lactate has evolved from a waste product into a central immunosuppressive regulator. Oncogenic KRAS and BRAF mutations drive aerobic glycolysis, causing glucose deprivation and massive lactate accumulation in the tumor microenvironment. Lactate suppresses immunity through three parallel mechanisms. It signals via GPR81 to recruit polymorphonuclear myeloid-derived suppressor cells (PMN-MDSCs) and inhibit T-cell function. It contributes to histone H3K18 lactylation, which silences effector genes including IFN-γ and GZMB while upregulating PD-L1 expression. It also acidifies the microenvironment to pH 6.0–6.5, directly impairing NK and T-cell activity. Concurrent lipid abundance stabilizes the MCT4 lactate exporter, forming a bidirectional feed-forward loop that amplifies lactate effects. Spatial metabolic heterogeneity creates distinct immune battlefields, with a supportive ‘metabolic oasis’—a concept proposed in this review—at the invasive front and a deeply immunosuppressive core. Thus, lactate acts as an epigenetic and signaling hub that bridges oncogenic mutations, metabolic competition and immune evasion. Targeting lactate metabolism through LDHA or MCT4 inhibition, modulation of histone lactylation, or disruption of lactate-lipid crosstalk, when combined with classical immune checkpoint blockade and guided by spatial biomarkers, offers a promising strategy to overcome immunotherapy resistance in this challenging subtype.

## 1. Introduction

Colorectal cancer (CRC) remains one of the most lethal malignancies worldwide, and its resistance to immune checkpoint inhibitors (ICIs), particularly in the microsatellite-stable (MSS) subtype, which accounts for 85–95% of cases, represents a major clinical challenge [1,2]. This resistance is not merely due to classical protein-based checkpoints but is fundamentally rooted in the metabolic landscape of the tumor microenvironment (TME), where CRC cells aggressively reprogram their metabolism to outcompete infiltrating immune cells for limited nutrients [3].

The Warburg effect, driven by oncogenic mutations in KRAS, BRAF, and MYC, enables CRC cells to consume glucose at rates far exceeding normal tissues, creating steep glucose gradients and severe nutrient deprivation [4]. However, the true consequence of this metabolic hijacking is not simply starvation; it is the massive production of lactate. In the CRC TME, lactate concentrations in tumor tissues homogenates have been reported to reach up to 40 µmol/g wet weight. Given that the tumor extracellular volume fraction is approximately 20–30%, this tissue content corresponds to an interstitial fluid concentration of >20 mM in necrotic regions, effectively transforming this glycolytic end-product from a metabolic waste into a potent immunosuppressive agent [1,5]. Lactate exerts its effects through three interconnected pathways. First, it acts as a signaling molecule via the GPR81 (HCAR1) receptor, promoting the recruitment of PMN-MDSCs and suppressing T-cell proliferation and cytokine production [6,7,8]. Second, lactate contributes to histone lactylation, an emerging epigenetic mark, which upregulates PD-L1 expression, silences effector genes such as IFN-γ and GZMB in CD8^+^ T cells, and polarizes tumor-associated macrophages towards an M2-like phenotype [9]. Third, lactate accumulation acidifies the TME to pH 6.0–6.5, directly impairing NK and T-cell function while further upregulating PD-L1 [10]. These mechanisms coalesce into a self-reinforcing loop that underpins the “cold” immune phenotype of MSS CRC [11,12].

Critically, this lactate-driven immunosuppression is amplified by concurrent lipid abundance [13,14,15]. Cholesterol and fatty acids stabilize the MCT4 lactate exporter, enhancing its efflux capacity, while lactate itself, through histone lactylation, upregulates lipogenic genes such as SREBF1 and FASN, creating a bidirectional feed-forward loop [16]. This metabolic crosstalk implies that targeting lactate alone may be counteracted by compensatory lipid-driven pathways, necessitating combination strategies that disrupt both lactate export and lipogenesis [1,17].

Thus, lactate has evolved from a disregarded metabolite to a central regulator of immune evasion in CRC, linking oncogenic mutations, epigenetic reprogramming, and microenvironmental acidification [18,19]. Understanding this integrated network offers new opportunities to overcome immunotherapy resistance, particularly in the MSS subtype, where metabolic checkpoints represent a promising but underexplored frontier [3,15,20]. This is a narrative review aiming to synthesize current understanding of lactate-driven immunosuppression in the CRC microenvironment. The relevant literature was identified through searches of PubMed and Web of Science databases up to July 2026, using keywords including ‘lactate,’ ‘colorectal cancer,’ ‘tumor microenvironment,’ ‘histone lactylation,’ ‘GPR81,’ ‘metabolic checkpoint,’ and ‘immunotherapy resistance.’ Priority was given to original research articles, systematic reviews, and high-impact mechanistic studies, with additional references identified from the bibliographies of retrieved articles. This narrative review synthesizes current understanding of lactate-driven immunosuppression in the CRC microenvironment, with a particular focus on the MSS subtype. Compared with existing reviews, this work provides three distinctive contributions: (1) it integrates the emerging concept of histone lactylation as an epigenetic checkpoint linking metabolism to immune evasion; (2) it proposes a lactate–lipid feed-forward loop that amplifies immunosuppression; (3) it introduces a spatial ‘metabolic oasis’ framework to explain the heterogeneous immune landscapes within the same tumor. These perspectives offer new insights into overcoming immunotherapy resistance in MSS CRC.

## 2. The Resource Economics of Metabolic Competition: The “Hunger Games” Between Tumor and Immune Cells

The tumor microenvironment (TME) is fundamentally a resource-limited, demand-excessive “metabolic battlefield.” CRC cells dramatically increase glucose uptake and glycolytic flux through the Warburg effect, preferentially relying on glycolysis for ATP production even under normoxic conditions [21]. This metabolic reprogramming not only sustains rapid tumor proliferation but also profoundly reshapes the nutrient landscape of the TME—the intense competition between tumor cells and infiltrating immune cells for finite metabolic resources constitutes a central mechanism of immune evasion and immunotherapy resistance [22,23].

### 2.1. Glucose Sequestration: The Engine Driving Lactate Flood

The most prominent metabolic hallmark of CRC is the full activation of aerobic glycolysis—the Warburg effect—driven by oncogenic mutations in KRAS, BRAF, and MYC. CRC cells upregulate glucose transporters (GLUT1) and rate-limiting glycolytic enzymes (HK2, LDHA), enabling glucose consumption at rates far exceeding those of normal tissues. This metabolic greed establishes a steep glucose concentration gradient within the TME:core regions experience markedly reduced glucose availability, compared with approximately 5 mM in normal serum [21,24]. Tumor cells, equipped with low-Km, high-V_max_ uptake kinetics, effectively outcompete neighboring cells for this limiting resource.

This glucose plundering exerts a dual consequence. On the immune side, infiltrating CD8^+^ effector T cells—which rely heavily on glycolysis upon activation—face abrupt fuel deprivation, leading to PEP depletion, mTORC1 inhibition, and impaired TCR signaling [23,24]. In contrast, regulatory T cells (Tregs), which can switch to fatty acid oxidation (FAO), display relative metabolic resilience under glucose-limited conditions [25]. On the tumor side, however, the confiscated glucose carbon skeletons are not efficiently channeled into the TCA cycle for oxidative phosphorylation; instead, through LDHA catalysis, a substantial proportion of glycolytic flux is converted into lactate [26]. As a result, while tissue lysates from necrotic tumor cores show lactate levels up to 40 µmol/g wet weight (reflecting the total cellular and extracellular pools), interstitial microdialysis studies indicate that the actual free lactate concentration bathing immune cells in these same regions readily surpasses 20 mM—a value far exceeding that of normal mucosatumor. Thus, the metabolic competition is far more than a simple “starvation game”—its terminal consequence is not merely ATP shortage but a lactate deluge that will reshape the entire immune landscape, as detailed in the following sections [13].

### 2.2. A Metabolic “Racing” Model: Quantifying the Metabolic Demands of Different Cell Types

**Figure 1:**
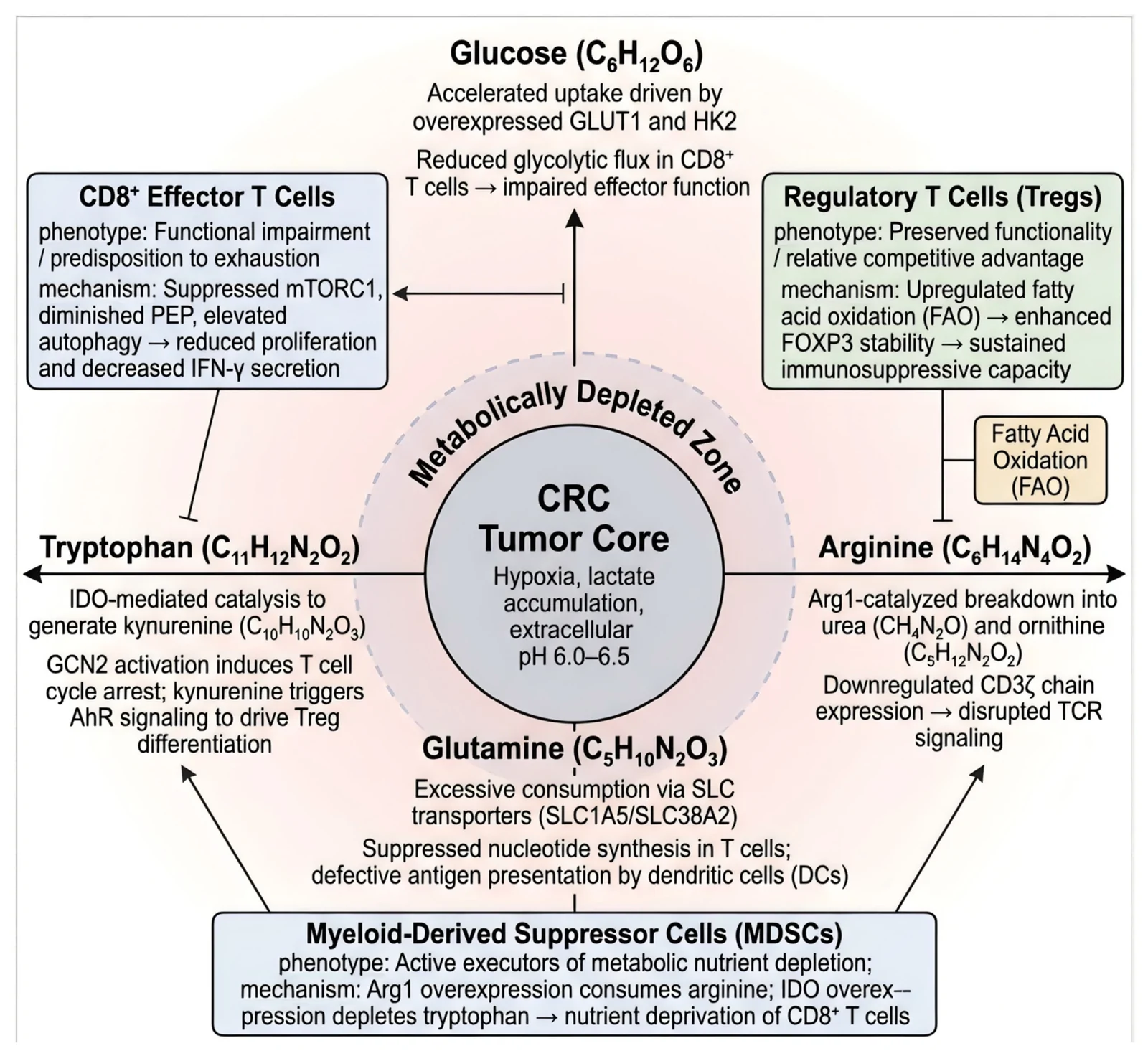
Schematic diagram depicting metabolic competition in the CRC tumor microenvironment. Four key nutrients—glucose, glutamine, tryptophan, and arginine—are depleted in the tumor core, impairing CD8^+^ T-cell metabolism, TCR signaling, and effector function, while conferring competitive advantages to immunosuppressive populations (Tregs and MDSCs). Created with Figdraw (www.figdraw.com).

Different cell types within the TME exhibit fundamental differences in nutrient uptake efficiency, metabolic preferences, and tolerance to deprivation, and these differences determine their relative “fitness” in the metabolic competition (Figure 1) [25,27,28].

CRC cells gain a dominant competitive position through oncogene-driven metabolic advantages. Mutations in KRAS, TP53, MYC, and EGFR significantly enhance the “cellular fitness” of CRC cells, making them “winner” cells in competition with neighboring cells [21,29,30,31].

Naïve T cells primarily rely on oxidative phosphorylation (OXPHOS) to meet energy demands at rest. However, once activated, CD8^+^ T cells undergo profound metabolic reprogramming, rapidly upregulating GLUT1 and shifting to aerobic glycolysis to support rapid proliferation, differentiation, and cytotoxic function. This metabolic switch renders CD8^+^ T cells exquisitely sensitive to glucose concentration—when glucose is massively consumed by tumor cells in the TME, glycolytic flux in CD8^+^ T cells collapses, and their effector functions become severely impaired. In CRC, highly glycolytic tumor cells effectively “outcompete” CD8^+^ T cells for glucose [23,24].

Unlike CD8^+^ T cells, Treg cells display unique metabolic flexibility. Treg cells not only utilize glycolysis but also rely heavily on fatty acid oxidation (FAO) and fatty acid synthesis (FAS) to meet energy demands and maintain FOXP3 stability. In the glucose-deprived TME, this metabolic flexibility confers a decisive competitive advantage on Treg cells. Studies show that Treg cells can gain a metabolic edge over conventional T cells by combining glycolysis with FAS/FAO, thereby outcompeting cells that rely predominantly on glycolytic flux. Consequently, under glucose-limited conditions, the relative fitness of Treg cells actually increases, which may help explain why Treg cells are often enriched in “cold” tumors [23,25].

Synthesizing the above differences, we propose a “metabolic fitness” framework: in the resource-competition landscape of the TME, a cell’s metabolic fitness is determined by its metabolic flexibility, substrate uptake efficiency, and tolerance to adverse conditions. CRC cells, driven by oncogenic mutations, achieve the highest metabolic fitness—they can efficiently plunder glucose while surviving hypoxia and acidosis. Treg cells rank second, owing to their FAO-based metabolic flexibility. CD8^+^ T cells, despite their high glycolytic rate upon activation, are at the most disadvantageous position because of their dependence on glucose concentration. This hierarchical gradient of metabolic fitness fundamentally shapes the functional landscape of immune cells in the TME. The metabolic demands of different immune subsets are summarized in Table 1.

### 2.3. Immunological Consequences of Metabolic Competition

Nutrient competition is not merely a matter of “starvation”—the scarcity of metabolic resources profoundly reprograms the functional state of immune cells through a series of signaling pathways, converting metabolic stress into a molecular switch for immunosuppression.

mTORC1 serves as the core metabolic sensor governing T-cell differentiation and function. Under glucose-replete conditions, mTORC1 maintains high activity, driving CD8^+^ T-cell differentiation into effector cells (Teff) and supporting their glycolytic metabolism. When glucose is massively consumed by tumor cells in the TME, mTORC1 signaling is suppressed [23,25]. Reduced mTORC1 activity directly impairs Teff proliferation and effector function. In parallel, Treg cells—which are less dependent on mTORC1 and capable of sustaining function via FAO—gain a relative survival advantage under glucose deprivation. This differential sensitivity orchestrates a shift from a pro-inflammatory to an anti-inflammatory immune landscape [23,25].

Phosphoenolpyruvate (PEP) is a glycolytic intermediate that sustains TCR-mediated Ca^2+^-NFAT signaling by inhibiting sarco/endoplasmic reticulum Ca^2+^-ATPase (SERCA). When glucose is limited, glycolytic flux declines, reducing PEP production. This relieves SERCA inhibition, leading to excessive ER Ca^2+^ depletion, disrupted Ca^2+^ signaling, and ultimately impaired NFAT-dependent TCR signal transduction [24].

Autophagy is a cellular survival mechanism that recycles nutrients and energy by degrading intracellular components during nutrient starvation. In the TME, persistent glucose and amino acid deprivation potently induces autophagy in T cells. Although short-term autophagy may help T cells survive under stress, chronic overactivation of autophagy has been associated with T cells towards functional exhaustion. Sustained autophagy not only accelerates T-cell metabolic failure but also promotes the transition to an exhausted phenotype (expressing inhibitory receptors such as PD-1 and TIM-3) through multiple mechanisms. Furthermore, nutrient-deprivation-induced autophagy is closely linked to mitochondrial dysfunction and accumulation of oxidative stress in T cells, further exacerbating functional decline [22].

A sobering caveat to the “metabolic competition” model is that most evidence comes from static co-culture systems or genetically engineered mouse models that poorly recapitulate the dynamic flux of human colorectal cancer. The tumor–immune relationship is not a zero-sum struggle for glucose; metabolic symbiosis frequently occurs, with certain tumor subclones feeding on lactate and tumor-associated macrophages scavenging lipids to nourish regulatory T cells. Moreover, the direction of causality remains ambiguous—infiltrating CD8^+^ T cells themselves can upregulate tumor glycolytic enzymes (e.g., LDHA) as a counter-response, meaning that the observed glucose depletion may partly reflect immune-driven metabolic resistance rather than pure tumor monopoly. Even more troubling is the phenomenon of epigenetic memory: after prolonged glucose restriction, T cells acquire irreversible H3K27me3 deposition at effector loci such as IFNG, so that subsequent nutrient replenishment fails to restore function. Thus, treating metabolic competition as a reversible “hunger game” underestimates its permanent imprint, and interventions must be deployed before T-cell fate is epigenetically cemented [11,23,25].

Together, the glucose sequestration and its downstream signaling derangements depict a TME where nutrients are monopolized, and immune cells struggle to survive. Yet this narrative remains incomplete unless we recognize that the glycolytic end-product—lactate—is not an inert waste awaiting clearance. When local lactate concentration reaches high millimolar levels (e.g., approximately 15 mM), it transforms from a bystander metabolite into an active offensive weapon [32]. In the following section, we delineate how this lactate flood, spawned directly by the competitive glucose hoarding, executes immunosuppression through three parallel yet interconnected pathways: GPR81-mediated signaling, histone lactylation, and microenvironmental acidification. In other words, while tumor cells “steal the food,” they simultaneously forge “chemical munitions” from their own metabolic refuse—and this complete causal chain positions lactate as the missing link that connects metabolic competition to immune evasion.

Beyond immediate signaling derangements, prolonged nutrient deprivation also triggers a slower but equally damaging process—autophagy. While transient autophagy may serve as a survival mechanism under acute stress, persistent autophagy upregulation—driven by sustained glucose and amino acid deprivation—accelerates T-cell functional exhaustion by promoting mitochondrial dysfunction and upregulating inhibitory receptor expression (PD-1, TIM-3) [27]. This autophagic exhaustion complements the metabolic competition effects described above, locking T cells into an irreversible dysfunctional state.

## 3. Lactate: From Metabolic Waste to a Signaling Hub of Immune Suppression

Lactate has undergone a remarkable re-evaluation in cancer biology—from a discarded glycolytic byproduct to a central signaling molecule that orchestrates metabolic, epigenetic, and immunological reprogramming in the tumor microenvironment [32,33,34]. In colorectal cancer, lactate is not merely a terminal product of the Warburg effect; it is an active driver of immune evasion, therapeutic resistance, and tumor progression [35,36]. This section dissects the multifaceted roles of lactate in CRC, focusing on its generation, its signaling functions via GPR81, its recently uncovered epigenetic activity through histone lactylation, and its contribution to acidic microenvironmental immunosuppression.

### 3.1. Genetic Hardwiring: Ensuring Glucose Flux Toward Lactate

The aerobic glycolysis described above (Section 2.1) is not a passive metabolic adaptation but is actively hardwired into CRC cells by driver mutations. Here we highlight two genetic features that specifically lock glucose flux toward lactate production rather than mitochondrial oxidation.

First, mutation-specific upregulation of LDHA: In isogenic CRC cell lines, KRAS G13D and BRAF V600E mutations not only upregulate HK2 but also stabilize HIF-1α, which post-transcriptionally increases LDHA activity by more than twofold, ensuring that pyruvate is preferentially reduced to lactate [30,37].

Second, hypoxia-amplified feedforward loops: Under hypoxic conditions, miR-210 represses ISCU (iron-sulfur cluster assembly enzyme), further suppressing TCA cycle activity and increasing lactate production by an additional 25% [38]. Concurrently, MCT4—the primary lactate exporter—is transcriptionally upregulated, facilitating the “dumping” of intracellular lactate into the microenvironment [39]. This genetic-hypoxic coupling establishes a dominant metabolic state in which lactate overproduction becomes a prevailing feature of CRC.

#### MSS-Specific Metabolic Hardwiring: KRAS/BRAF-Driven Lactate Production

The clinical relevance of lactate-driven immunosuppression is particularly pronounced in microsatellite-stable (MSS) colorectal cancer, which constitutes approximately 85–95% of all CRC cases and remains refractory to immune checkpoint inhibitors [2]. MSS CRC is distinguished by a high prevalence of oncogenic driver mutations—KRAS mutations occur in approximately 30–50% of all CRC cases, and BRAF V600E mutations in approximately 5–15%—that directly hardwire the metabolic program toward aerobic glycolysis and lactate overproduction(Table 2) [37,40,41].

Mechanistically, mutant KRAS has been associated with lactate production through at least three convergent pathways. First, KRAS activates ERK1/2, which phosphorylates TRIP6 and triggers its dissociation from KDM1A; this disruption enhances ENO2 transcription via KDM1A-mediated demethylation of H3K9me1/2, culminating in elevated glycolysis and lactate secretion [1,13,42]. Second, KRAS stabilizes HIF-1α, which transcriptionally upregulates both CSF2 and glycolytic genes, creating a feedforward loop that amplifies lactate output [1,42]. Third, KRAS mutations directly increase intracellular lactate pools, which serve as substrates for histone lactylation—notably, mutant KRAS elevates H3K9 lactylation (H3K9la) levels, and this modification is associated with poor prognosis and advanced clinical stages in KRAS-mutant CRC patients [3]. Similarly, BRAF V600E-driven metabolic reprogramming shows a dose-dependent relationship: glucose consumption and lactate production are proportional to BRAF V600E protein levels, confirming that the mutant protein itself dictates glycolytic flux [43].

Importantly, these mutation-driven metabolic alterations are not merely epiphenomena but actively reinforce immune evasion. The lactate generated through KRAS/BRAF-driven glycolysis fuels histone lactylation at the promoters of immunosuppressive genes such as PD-L1 and VEGFA, creating a self-sustaining cycle: oncogenic mutation → glycolysis → lactate → histone lactylation → immune checkpoint upregulation → further immune escape [13]. This mutation-lactylation-immunosuppression axis represents a MSS-specific vulnerability that distinguishes CRC from MSI-H tumors, where hypermutation rather than metabolic rewiring dominates the immune landscape. Therefore, targeting lactate production at the level of mutant KRAS/BRAF signaling—rather than merely inhibiting downstream LDHA—may offer a more MSS-selective therapeutic strategy.

While lactate-driven immunosuppression is a shared feature across many solid tumors, its pathogenic impact in MSS CRC is amplified by mutation-driven metabolic rewiring (KRAS/BRAF) and CAF-mediated stromal barriers, which together establish a metabolically hostile and immune-excluded microenvironment.

### 3.2. Lactate as a Signaling Molecule: The GPR81 Receptor Pathway

Beyond its metabolic role, lactate functions as a bona fide signaling molecule through the G-protein-coupled receptor GPR81 (also known as HCAR1, hydroxycarboxylic acid receptor 1). In CRC, lactate activates GPR81 on both tumor cells and infiltrating immune cells, triggering downstream cascades that profoundly reshape the TME.

On CRC cells, GPR81 activation induces the secretion of chemokines CCL2 and CCL7, which recruit CCR2^+^ polymorphonuclear myeloid-derived suppressor cells (PMN-MDSCs). These PMN-MDSCs potently suppress CD8^+^ T-cell activation, creating a barrier to anti-tumor immunity. In *Hcar1*-knockout mice, CRC tumor burden is significantly reduced, and CD8^+^ T-cell infiltration significantly increased, directly implicating GPR81 as a critical mediator of lactate-induced immunosuppression.

On immune cells, GPR81 signaling inhibits the cAMP/PKA pathway, which is essential for T-cell activation and effector function. Lactate-mediated suppression of this pathway impairs T-cell proliferation and cytokine production (IFN-γ, TNF-α). In tumor-associated macrophages (TAMs), lactate–GPR81 signaling promotes M2-like polarization, shifting macrophages toward a pro-tumor, immunosuppressive phenotype that secretes IL-10 and TGF-β [44]. This polarization is further amplified by mutant KRAS, which stabilizes HIF-1α and increases CSF2 and lactate production, together reprogramming macrophages to a TAM-like phenotype that promotes cetuximab resistance [45]. Therapeutically, reserpine—an FDA-approved drug—inhibits HCAR1 activation, reducing CCR2^+^ PMN-MDSC recruitment and enhancing CD8^+^ T-cell activity; in CRC mouse models, reserpine combined with anti-PD-1 therapy significantly suppresses tumor growth [46].

### 3.3. Histone Lactylation: Metabolic–Epigenetic Crosstalk

Of note, the H3K9la mark associated with KRAS mutations is distinct from the H3K18la modification described in the following section. While H3K9la appears to be specifically driven by mutant KRAS signaling and linked to cholesterol metabolism gene expression, H3K18la represents a broader lactate-induced epigenetic mark that regulates diverse immunosuppressive and metabolic genes across multiple cell types within the TME.

In CRC, lactate accumulation has been associated with H3K18 lactylation at the promoters of key genes, directly linking metabolic flux to epigenetic reprogramming [47]. For example, lactate promotes H3K18 lactylation at the PD-L1 promoter, enhancing PD-L1 expression and thereby suppressing CD8^+^ T-cell cytotoxicity [48]. Lactate also upregulates genes involved in proliferation (c-Myc, cyclin D1) and survival through H3K18 lactylation [49]. In hypoxic CRC cells, HIF-1α upregulates LDHA, increasing lactate production and H3K18 lactylation at the *RUBCNL* promoter, which enhances autophagosome maturation and supports cell survival under hypoxia; in bevacizumab-resistant CRC patients, H3K18 lactylation levels are twofold higher than in sensitive patients, correlating with poor overall survival.

Lactylation also affects immune cells. In myeloid cells, lactate-induced H3K18 lactylation upregulates METTL3, an m^6^A methyltransferase, which mediates m^6^A modification of *JAK1* mRNA, increasing JAK1 protein translation and activating the STAT3 pathway—thereby promoting M2 polarization of TAMs [27,48]. In CD8^+^ T cells, histone lactylation silences effector genes such as *IFN-γ* and *GZMB*, contributing to T-cell exhaustion [27]. The gut microbiota *Fusobacterium nucleatum* further amplifies this axis: it increases lactate accumulation in CRC, promoting H3K18 lactylation at the PD-L1 promoter and increasing PD-L1 expression, thereby suppressing CD8^+^ T-cell activity; in Fn-positive CRC patients, PD-L1 expression is twofold higher and prognosis poorer [48].

Importantly, histone lactylation is dynamically reversible, regulated by “writers” and “erasers” (acetyltransferases and deacetylases that also recognize lactyl-CoA or remove lactyl groups). This reversibility offers therapeutic opportunities: HDAC inhibitors such as panobinostat reduce H3K18 lactylation and downregulating PD-L1 expression in CRC cells; when combined with JQ1 (a BRD4 inhibitor), these agents enhance CD8^+^ T-cell infiltration and suppress tumor growth in preclinical models [50,51]. Sulconazole, an antifungal drug, inhibits glycolysis, reducing lactate production and histone lactylation at the PD-L1 promoter, and restoring T-cell function [52].

### 3.4. Lipid Milieu Amplifies Lactate Signaling

The epigenetic and signaling functions of lactate do not operate in isolation; they are profoundly modulated by the concurrent lipid-rich nature of the CRC TME. Recent evidence indicates that cholesterol and free fatty acids directly enhance the lactate export machinery [1,3,53]. Mechanistically, cholesterol incorporation into lipid rafts stabilizes the MCT4/CD147 complex at the plasma membrane, reducing MCT4 internalization and prolonging its half-life, which enhances net lactate efflux under hyperlipidemic conditions compared with lipid-poor environments [3,13]. Fatty acids have also been associated with upregulated MCT4 expression and enhanced lactate secretion in CRC cells [1,13]. This lipid-driven amplification creates a vicious feedforward loop: lactate accumulation promotes histone lactylation and lipogenic gene expression (as detailed in Section 4.5), which enriches the TME with lipids, and these lipids in turn stabilize MCT4 to pump out even more lactate [54]. Consequently, tumors situated in obese or high-fat-diet hosts may experience exacerbated lactate-mediated immunosuppression, suggesting that host metabolic status directly shapes the efficacy of lactate-targeted therapies. This crosstalk also implies that combining lactate-neutralizing strategies with lipid-lowering agents (e.g., statins) could synergistically dismantle this bidirectional amplification circuit [3,13,53,55].

### 3.5. Acidic Microenvironment and Its Immunosuppressive Effects

Lactate accumulation does more than signal and epigenetically modify—it directly acidifies the TME. The massive lactate production by CRC cells promotes extracellular pH down to 6.0–6.5, a condition that profoundly impairs immune cell function [1,3,13,56].

Acidosis suppresses natural killer (NK) cells and T cells by multiple mechanisms. It inhibits glycolytic enzymes such as phosphofructokinase, disrupts TCR signaling and activation marker expression, and diminishes NK cytotoxic granule release. Furthermore, acidification directly upregulates PD-L1 expression on tumor cells and TAMs, reinforcing the immune checkpoint axis [53].

**Figure 2:**
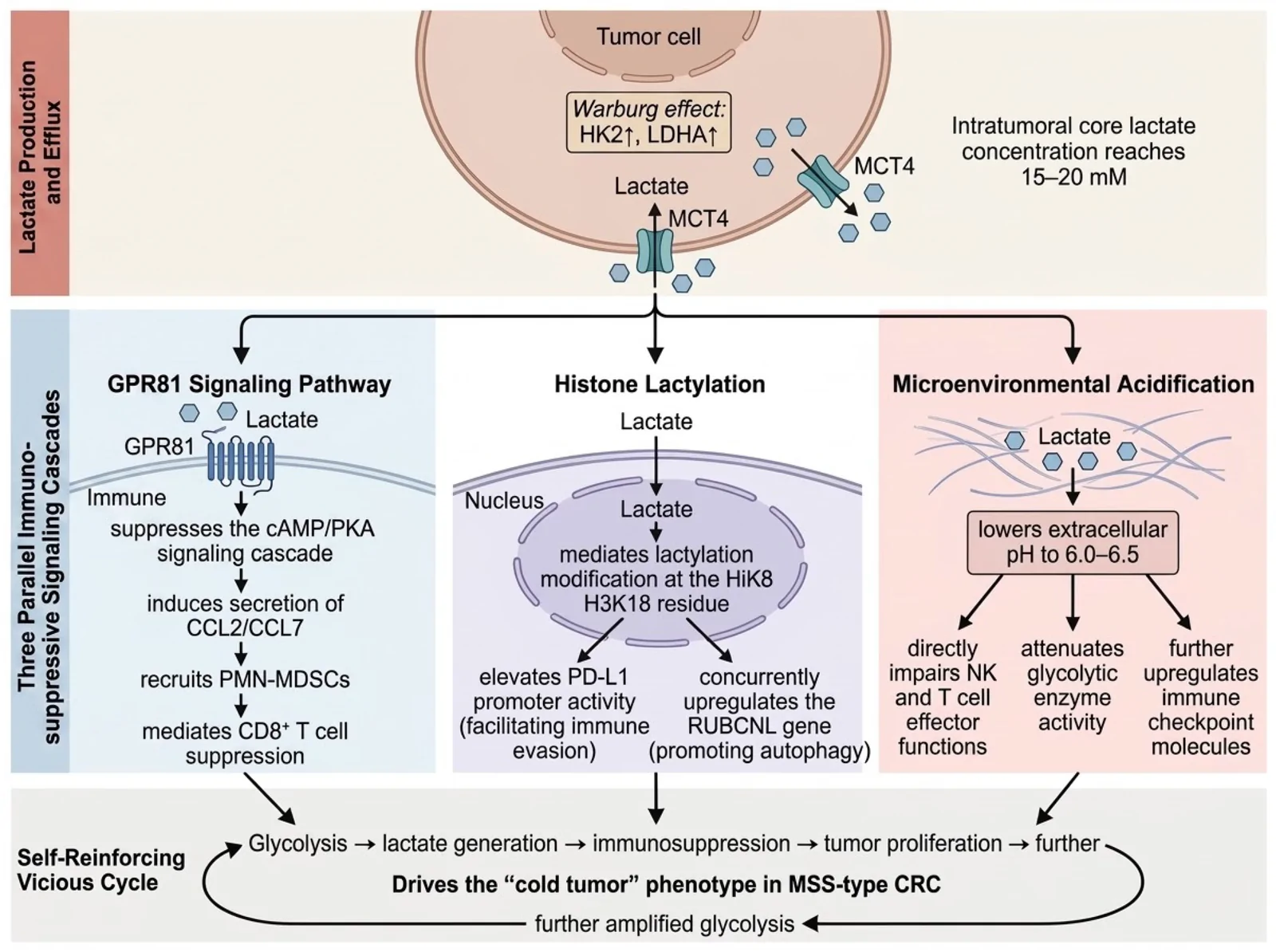
Schematic illustration of the three parallel immunosuppressive mechanisms of lactate in the CRC microenvironment: GPR81/HCAR1 signaling, histone H3K18 lactylation, and microenvironmental acidification. These pathways converge to suppress CD8^+^ T-cell and NK-cell function, upregulate PD-L1 expression, and drive a self-reinforcing cycle that sustains the “cold tumor” phenotype in MSS CRC. Arrows denote causal or sequential relationships between depicted components. Created with Figdraw (www.figdraw.com).

The combined effect of lactate’s three immunosuppressive channels—GPR81 signaling, histone lactylation, and acidification—creates a self-reinforcing loop: lactate produced by glycolysis suppresses immunity through parallel pathways, while immune suppression permits further tumor growth and lactate production [1,53]. This vicious cycle underpins the “cold” immune phenotype of many CRC, particularly MSS tumors, and highlights lactate metabolism as a critical bottleneck for effective immunotherapy as summarized in Figure 2 [13]. The triple immunosuppressive mechanisms of lactate in CRC are summarized in Table 3.

## 4. Lipid Metabolism: The “Fate Regulator” of Immune Cell Differentiation

The differential metabolic sensitivity between CD8^+^ effector T cells and Tregs, briefly introduced in Section 2.3, becomes further pronounced in the lipid-rich CRC TME. abundant fatty acids not only sustain FAO-dependent energy demands but also enhance FOXP3 stability and Treg survival [25,57,58]. This lipid-dependent survival advantage positions Tregs as formidable competitors in the metabolic landscape, and the detailed molecular wiring of this pathway is explored below. Lipid metabolism has emerged as a critical determinant of immune cell function and differentiation within the colorectal cancer microenvironment [54,55,59]. Beyond serving as structural membrane components and energy stores, lipids—particularly cholesterol and fatty acids—act as potent signaling molecules that reshape the metabolic fitness and functional polarity of infiltrating immune cells [56,60,61]. In CRC, the interplay between tumor-driven lipid accumulation and immune cell lipid handling creates a complex regulatory network that fundamentally influences antitumor immunity and responsiveness to immunotherapy [62,63,64].

### 4.1. Lipid Accumulation and Cholesterol as a Metabolic–Immune Checkpoint

Epidemiological and preclinical evidence links high-fat diet and obesity to CRC initiation and progression. Obese individuals exhibit elevated circulating free fatty acids and cholesterol, which are taken up by CRC cells or synthesized de novo via upregulated fatty acid synthase (FASN). CRC cells upregulate FASN and other lipogenic enzymes, resulting in a TME rich in fatty acids and cholesterol—a milieu actively sculpted by tumor cells to fuel their own growth and manipulate immune cell behavior [65,66].

This lipid-rich environment has led to the proposal that cholesterol metabolism functions as a critical metabolic–immune checkpoint—a regulatory node that operates not through single receptor–ligand interactions but through systemic remodeling of the TME via alterations in membrane properties, bioactive lipid mediators, and intracellular signaling cascades. Tumor cells “hijack” cholesterol metabolism to achieve a dual effect: they fortify their own defensive “armour” against immune attack while simultaneously “blunting” the offensive weapons of immune cells. This hijacking involves upregulated cholesterol uptake and synthesis, enhanced esterification for storage, and reprogrammed efflux pathways—collectively establishing a cholesterol-based barrier to effective antitumor immunity [67,68].

### 4.2. Differential Regulation of Immune Cells by Lipids

Under physiological conditions, cholesterol is a cornerstone of T-cell activation and immunological synapse formation, supporting membrane microdomain organization and signaling. However, in the lipid-rich CRC TME, excessive free cholesterol is esterified by acyl-CoA cholesterol acyltransferase 1 (ACAT1) and stored as cholesterol esters in lipid droplets. This accumulation triggers lipotoxicity, leading to mitochondrial dysfunction, endoplasmic reticulum stress, and ultimately functional exhaustion of CD8^+^ T cells [69]. Paradoxically, while cholesterol esters accumulate intracellularly, the plasma membrane—which requires free cholesterol for proper signaling—experiences a state of cholesterol “starvation” because the esterification traps cholesterol away from membranes [70]. This sequestering effectively “locks” T-cell activity, suppressing TCR signaling and cytokine production. Notably, the above ‘membrane starvation’ model coexists with a complementary pathway: excessive free cholesterol accumulation in the ER and mitochondria directly triggers lipotoxicity and drives T-cell exhaustion. Thus, the subcellular distribution of cholesterol—rather than total cellular content—determines its functional impact, reconciling these two apparently opposing observations [70,71]. This spatiotemporal complexity underscores the need for precise, subcellular-level reprogramming of cholesterol distribution to restore T-cell function without exacerbating lipotoxicity.

**Figure 3:**
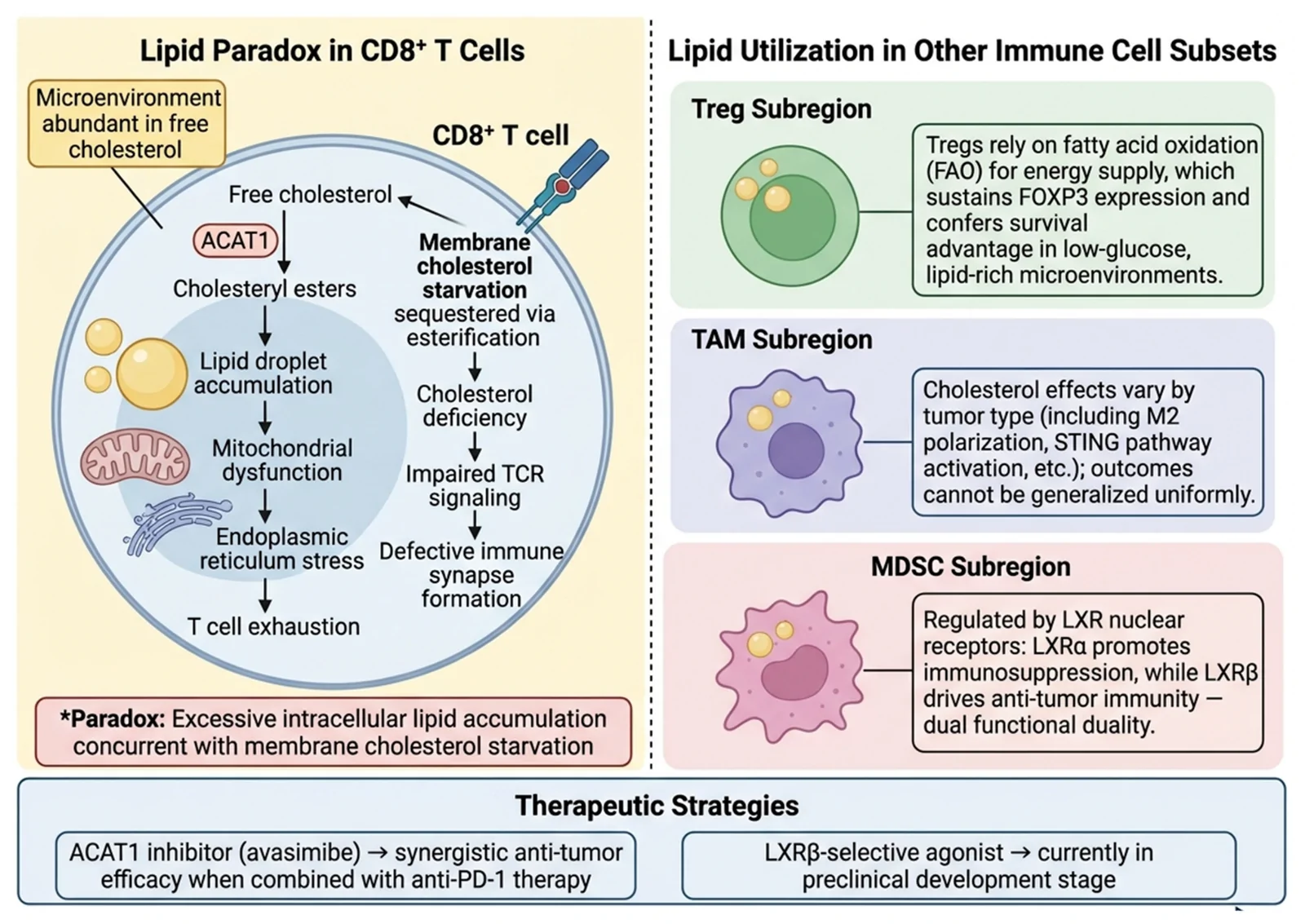
Lipid metabolism orchestrates immune cell fate determination in the colorectal cancer microenvironment. Figure 3 was created with Figdraw (www.figdraw.com).

Differential regulation of immune cells by lipids is illustrated in Figure 3. Regulatory T cells possess a distinct metabolic advantage in lipid-rich environments. Treg cells rely heavily on fatty acid oxidation (FAO) to meet their energy demands, and elevated fatty acid availability in the CRC TME enhances their survival and functional stability [72]. Moreover, cholesterol metabolism reinforces Treg-cell stability by promoting FOXP3 expression and sustaining their immunosuppressive capacity [73]. In the lipid-abundant TME, Treg cells are thus “armed” with metabolic fuels that allow them to outcompete effector T cells, effectively reinforcing immune suppression and contributing to the failure of immunotherapies.

In colorectal cancer, tumor-associated macrophages (TAMs) undergo lipid-driven functional reprogramming that similarly promotes immunosuppression. Recent evidence demonstrates that CRC TAMs exhibit enhanced fatty acid oxidation (FAO) via the DPP7-CPT1A axis, which promotes M2 polarization and CD8^+^ T-cell exhaustion [74]. Lactate-driven VSIG4 upregulation further enhances FAO through JAK2/STAT3-PPARγ signaling, sustaining the immunosuppressive phenotype [75]. Additionally, ACSL3 lactylation protects CRC TAMs from ferroptosis, maintaining their M2-like state [76]. These CRC-specific findings highlight that therapeutic targeting of TAM lipid metabolism must abandon a ‘one-size-fits-all’ approach and instead be tailored to the specific tumor microenvironment and macrophage polarization state.

In myeloid-derived suppressor cells (MDSCs), the two LXR isoforms exert opposing effects: LXRα activation exacerbates immunosuppression, whereas LXRβ activation enhances antitumor immunity. This isoform-specific functional dichotomy within the same signaling pathway highlights the necessity for “surgical-precision” interventions that selectively modulate LXRβ without activating LXRα, lest immunotherapy be inadvertently undermined [77,78]. Lipid metabolism differentially regulates immune cell subsets in CRC (Table 4).

### 4.3. Crosstalk Between Lipid Metabolism and Immune Checkpoints

The intersection between lipid metabolism and classical immune checkpoints offers promising therapeutic opportunities. Inhibition of ACAT1—the enzyme that esterifies cholesterol—has been shown to synergize with anti-PD-1 antibodies, enhancing CD8^+^ T-cell effector function and improving tumor control in preclinical models [69,70,71]. The ACAT1 inhibitor avasimibe, originally developed for cardiovascular indications, is being repurposed for cancer immunotherapy. Similarly, targeting cholesterol metabolism through other nodes—such as modulating LXR signaling or restricting lipid uptake—may overcome at least some forms of immunotherapy resistance, particularly in tumors with high lipid burden. These findings position lipid metabolism as an attractive adjunctive target for combination regimens designed to “re-arm” immune cells within the metabolically hostile CRC TME [75].

While lipids are rightly highlighted as immune-fate determinants, therapeutic exploitation remains hindered by several unaddressed realities. ACAT1 inhibition, though synergistic with anti-PD-1 in preclinical models, carries substantial off-target risks because ACAT1 maintains cholesterol homeostasis in adrenal glands and intestinal epithelia; chronic blockade may impair steroidogenesis and provoke colitis [80]. Tumor-associated macrophages display far greater heterogeneity than the M1/M2 dichotomy implies—even within a single CRC, marginal and core TAMs exhibit divergent lipid uptake and oxidation capacities, rendering a uniform anti-lipogenic approach futile. The promise of LXRβ-selective agonists remains unfulfilled owing to the lack of orally bioavailable compounds and the overlapping regulatory roles of LXRα/β in hepatic cholesterol reverse transport [1,81,82]. Moreover, the reversibility of lipid-induced T-cell exhaustion is questionable; cholesterol ester accumulation causes mitochondrial damage that may become epigenetically fixed, so that simply removing the lipid overload might not restore effector function. Future combination regimens should therefore pair lipid-lowering agents (e.g., statins) with mitophagy inducers, and must be tailored to the spatial and subset-specific lipid profiles of individual tumors [83].

### 4.4. CAF-Mediated Metabolic Barriers and T-Cell Exclusion in MSS CRC

The metabolic–immunological landscape of MSS CRC is profoundly shaped by cancer-associated fibroblasts (CAFs), which are disproportionately enriched in the desmoplastic stroma of MSS tumors compared with MSI-H CRC [84,85]. CAFs contribute to immunosuppression through at least three interconnected metabolic pathways that together establish a “metabolic barrier” against T-cell infiltration and function [86].

First, CAFs undergo a pronounced shift toward aerobic glycolysis—a process termed the “reverse Warburg effect”—producing high levels of lactate and pyruvate that are shuttled to adjacent CRC cells to fuel oxidative phosphorylation and anabolic biosynthesis. This CAF-derived lactate accumulation acidifies the TME, enhances extracellular matrix remodeling, and directly suppresses cytotoxic T-cell activity. Notably, CAFs are not merely passive lactate producers; they also utilize extracellular lactate to promote their own activation and functions, establishing a self-amplifying metabolic loop [87].

Second, CAFs secrete a portfolio of immunosuppressive metabolites—including arginase, polyamines, and lactate—that directly inhibit cytotoxic T-cell function. This metabolic crosstalk extends to tumor-associated macrophages (TAMs): CAF-derived lactate promotes M2 polarization, while TAM-secreted cytokines reciprocally enhance glycolysis in CRC cells, creating a feedforward immunosuppressive circuit [88].

Third, CAFs collaborate with the extracellular matrix to erect a physical–metabolic “exclusion barrier.” In MSS CRC, CAF-derived chondroitin-6-sulfate (C-6-S) interacts with M2 macrophages at the invasive margin, forming a barrier that excludes T cells. Mechanistically, C-6-S promotes co-nuclear translocation of pSTAT3 and GLI1, activating the JAK/STAT3 and Hedgehog pathways. This C-6-S-induced immune exclusion has been defined as an “immunometabolic checkpoint”—a concept that bridges the classical immune checkpoint framework with metabolic regulation (discussed further in Section 5). Importantly, C-6-S-targeted strategies have been shown to decrease M2 macrophage abundance, reprogram the immunosuppressive TME, and enhance anti-PD-1 responses in MSS CRC models [89].

The transcriptional regulator ASCL2 further amplifies this CAF-centered metabolic barrier. ASCL2 is overexpressed in MSS CRC and maintains a stemness phenotype accompanied by lower tumor-infiltrating lymphocyte density. ASCL2 overexpression increases TGFβ levels, which stimulate local CAF activation and induce an immune-excluded microenvironment; conversely, ASCL2 deletion reduces activated CAFs and increases CD8^+^ T-cell infiltration. Collectively, these findings position CAF-mediated metabolic reprogramming as a central, MSS-selective driver of immunotherapy resistance—one that operates through both metabolic metabolite secretion and physical stromal remodeling [90,91].

Notably, the metabolic and physical barriers erected by CAFs are not uniformly distributed throughout the tumor but exhibit spatial heterogeneity that mirrors the broader metabolic gradients described in Section 6. CAF density and activation status vary along the invasive front–tumor core axis, with the densest stromal barriers often localized at the invasive margin, where they physically exclude T cells from entering the tumor core. This spatial organization directly links CAF-mediated metabolic immunosuppression to the ‘metabolic oasis’ concept: in regions where CAFs are sparse and nutrient perfusion is relatively preserved (i.e., the invasive front), immune cells may retain functional capacity; conversely, where CAFs are abundant and metabolic waste accumulates (i.e., the tumor core), immune cells are excluded or rendered dysfunctional. Thus, CAF-driven metabolic barriers represent a key structural determinant of the spatial immune landscape in MSS CRC [84].

### 4.5. Lactate-Lipid Metabolic Crosstalk: Potentially Establishing a Self-Reinforcing Loop

Mechanistically, the lactate-driven histone H3K18 lactylation has been demonstrated to directly regulate lipid metabolism in cancer cells [92]. For instance, lactylation at the promoter regions of lipogenic genes, including SREBF1 and FASN, promotes their transcription, thereby upregulating de novo fatty acid synthesis and cholesterol esterification [93]. This epigenetic coupling demonstrates that lactate is not merely a terminal waste product but an initiator of lipogenic reprogramming, enabling tumor cells to simultaneously secure membrane building blocks and energy stores while extruding acid. Conversely, lipid abundance feeds back on lactate dynamics. Cholesterol enrichment in the plasma membrane enhances the clustering and stability of CD147—the chaperone of MCT4—thereby increasing MCT4 surface expression and lactate efflux capacity in lipid-rich microenvironments [94]. Furthermore, fatty acid oxidation (FAO) in tumor cells supplies acetyl-CoA to fuel histone acetylation and lactylation crosstalk, creating a permissive epigenetic state for further glycolytic gene expression. This lactate-lipid interlock implies that therapeutic strategies targeting only one arm (e.g., LDHA inhibition or ACAT1 blockade) may be counteracted by compensatory flux through the other. Therefore, future combination regimens should consider dual blockade of lactate export and lipogenesis, or exploit this crosstalk by using statins to disrupt lipid-dependent MCT4 membrane stabilization, thereby sensitizing CRC to metabolic–immune checkpoint combinations.

## 5. Metabolic Checkpoints: Novel Intervention Targets Bridging Metabolism and Immunity

### 5.1. The Concept of “Metabolic Checkpoints”

Classical immune checkpoints (PD-1, CTLA-4, TIM-3, LAG-3) are often limited in “cold” MSS CRC, where the metabolically hostile TME constrains effector lymphocytes even when checkpoint proteins are blocked. This has prompted recognition of a deeper regulatory layer: metabolic checkpoints. Unlike protein-based checkpoints, metabolic checkpoints operate at the level of nutrient availability and pathway flux, determining whether T cells possess the metabolic capacity to execute effector programs. Together, classical and metabolic checkpoints constitute a “metabolic–immune” double brake; breaking both may be necessary for effective immunotherapy [20,95,96].

#### MSS Metabolic Subtypes: The IMMETCOLS Framework

The IMMETCOLS classification stratifies MSS CRC into three metabolically distinct clusters with differential therapy responses (Table 5) [97].

All subtypes display metabolic plasticity under therapeutic pressure, suggesting adaptive rewiring as a core resistance mechanism. Subtype-guided strategies—LDHA/MCT inhibition for IMC1, lactate oxidation blockade for IMC3—offer a precision medicine roadmap to overcome the historical “one-size-fits-all” failure of immunotherapy in MSS CRC.

### 5.2. Key Metabolic Checkpoint Molecules

#### 5.2.1. The IDO–Tryptophan Axis

Indoleamine 2,3-dioxygenase (IDO) is the rate-limiting enzyme in tryptophan catabolism along the kynurenine pathway. IDO is upregulated in many tumors, including CRC, often in response to interferon-γ from infiltrating T cells—a feedback mechanism that converts an inflammatory signal into an immunosuppressive one. Tryptophan depletion by IDO arrests T-cell cycle progression in the G1 phase through the GCN2 stress-sensing kinase pathway, which inhibits mTORC1 signaling and reduces protein synthesis. Simultaneously, the accumulated kynurenine metabolites activate the aryl hydrocarbon receptor (AhR) on T cells and antigen-presenting cells, promoting the differentiation of naïve T cells into immunosuppressive Treg cells while suppressing Th17 and effector T-cell responses. This dual action—starvation plus signaling—makes the IDO–tryptophan axis a prototypical metabolic checkpoint. Although IDO inhibitors such as epacadostat have shown preclinical synergy with PD-1 blockade, clinical translation has been challenging, highlighting the complexity of targeting metabolic pathways without disrupting essential homeostasis [64,98,99]. Key metabolic checkpoints in the CRC microenvironment are listed in Table 6.

#### 5.2.2. The Arg1–Arginine Axis

Arginase 1 (Arg1) is highly expressed by myeloid-derived suppressor cells (MDSCs) and some tumor-associated macrophages in the CRC TME. Arg1 catalyzes the hydrolysis of L-arginine to urea and ornithine, thereby depleting the extracellular arginine pool. Arginine is an essential amino acid for T-cell activation and proliferation because it supports the expression of the CD3ζ chain of the TCR complex and sustains mTORC1 activity. Arginine deprivation also impairs T-cell memory formation and promotes T-cell exhaustion. Importantly, the Arg1–arginine axis synergizes with PD-1/PD-L1 signaling: PD-1 engagement further suppresses T-cell metabolism, and arginine starvation upregulates PD-L1 expression on tumor cells, creating a reinforcing loop. Preclinically, Arg1 inhibitors have been shown to restore T-cell function, and combination with checkpoint inhibitors is being explored in early-phase trials [100].

Lymphocyte-activation gene 3 (LAG-3) is an inhibitory receptor upregulated on exhausted T cells, and it binds to MHC class II molecules with higher affinity than CD4. Beyond its canonical inhibitory function, recent studies have revealed that LAG-3 signaling is intimately linked to T-cell metabolic state. LAG-3 engagement suppresses T-cell glycolysis and mitochondrial oxidative phosphorylation, reducing the metabolic fitness necessary for sustained effector function. Conversely, LAG-3 blockade can partially restore glucose uptake and glycolytic capacity in exhausted T cells, suggesting that LAG-3 acts as both a signaling checkpoint and a metabolic brake. This dual role positions LAG-3 as a bridge between classical and metabolic checkpoints, and anti-LAG-3 antibodies (e.g., relatlimab) are now being combined with PD-1 inhibitors in clinical trials for various cancers, including CRC.

T-cell immunoglobulin and mucin-domain containing-3 (TIM-3) is another exhaustion marker that is upregulated on dysfunctional T cells. Beyond its established role in inhibiting T-cell responses via galectin-9 and other ligands, TIM-3 has been linked to lipid metabolism. TIM-3 expression is elevated in T cells that have accumulated cholesterol, and cholesterol-rich microenvironments promote TIM-3 upregulation, contributing to an exhaustion-prone state. Mechanistically, TIM-3 may interfere with fatty acid oxidation and mitochondrial respiration, further compromising T-cell metabolism. This connection suggests that targeting lipid metabolism could indirectly modulate TIM-3 activity, and therapies that reduce cholesterol burden in T cells might synergise with TIM-3 blockade to rejuvenate antitumor immunity [71,72].

### 5.3. Metabolic Checkpoints as Combination Therapeutic Targets

The concept of metabolic checkpoints offers a rational framework for combination therapy. By targeting upstream metabolic pathways that constrain T-cell function, we may enhance the efficacy of classical immune checkpoint inhibitors and overcome resistance. Preclinical studies have demonstrated that IDO inhibitors (e.g., epacadostat) can synergise with PD-1 blockade, although clinical results have been mixed, possibly due to compensatory pathways. Arg1 inhibitors and LAG-3 antibodies are advancing into clinical exploration, and early data show promise in restoring T-cell metabolic fitness [101,102,103,104,105].

The concept of “metabolic checkpoints” offers an appealing framework, but its clinical translation is constrained by redundancy, toxicity, and inappropriate timing. IDO inhibitors failed in phase III trials partly because TDO compensates for IDO blockade and because tumors directly uptake exogenous kynurenine; similarly, Arg1 inhibition can be counterbalanced by NOS2 upregulation, as both enzymes compete for the same substrate L-arginine, and their functional balance determines the metabolic fate of arginine in the tumor microenvironment [106,107]. Systemically, IDO serves physiological roles in the placenta, central nervous system, and gut mucosa, so protracted inhibition may unleash autoimmunity or neurobehavioral abnormalities [108]. LAG-3 and TIM-3 are constitutively expressed on regulatory T cells, and their systemic blockade could break peripheral tolerance. Perhaps the most critical oversight is the sequence of intervention—most preclinical studies co-administer metabolic and immune checkpoint blockers, but if T cells have already entered terminal exhaustion with an epigenetically locked state, providing metabolic fuel alone cannot revive their effector program [109]. A more rational strategy would be a “metabolic preconditioning” phase (e.g., brief LDHA/MCT inhibition) followed by checkpoint blockade, allowing T cells to escape early metabolic domestication [110]. Equally pressing is the absence of validated biomarkers; serum lactate/pyruvate ratios, MCT4/PKM2 expression scores, or tumor lactate imaging must be prospectively tested to select patients who will truly benefit from metabolic–immune combinations.

In summary, metabolic checkpoints—exemplified by the IDO–tryptophan, Arg1–arginine, LAG-3–metabolism, and TIM-3–lipid axes—represent a new frontier in immunometabolism [100]. They are not merely alternative targets but integral components of the immune response that must be addressed alongside classical checkpoints to achieve durable antitumor immunity. The challenge ahead lies in identifying predictive biomarkers, optimizing combination regimens, and avoiding on-target toxicities in normal tissues that rely on these metabolic pathways. Nonetheless, the integration of metabolic checkpoint modulation into the CRC immunotherapy arsenal offers a scientifically grounded strategy to break the resistance barrier that has long limited success in this disease.

## 6. Spatial Metabolic Heterogeneity: “Terrain” and “Battlefields” Within the TME

### 6.1. Breakthroughs in Spatial Multi-Omics Technologies

The advent of spatial multi-omics technologies has revolutionized our ability to dissect the CRC TME at unprecedented resolution. Imaging mass cytometry (IMC), spatial transcriptomics, spatial proteomics, and spatial metabolomics (e.g., AFADESI-MSI) now enable simultaneous detection of dozens of proteins, transcripts, and metabolites while preserving their spatial coordinates within tissue architecture [111,112,113]. Unlike conventional bulk analyses, which average signals across heterogeneous cell populations, spatial metabolomics provides three-dimensional qualitative, quantitative, and positional information about metabolites in tissues. These technologies reveal not merely which cell types are present, but where they are located, how they interact, and what metabolic gradients they experience—transformative insights that are essential for understanding the functional consequences of metabolic competition in CRC [114].

### 6.2. Discovery of Spatial Niches in the CRC Microenvironment

A landmark study published in *Cell Discovery* identified a specific spatial niche that distinguishes immunotherapy responders from non-responders in CRC. In responding patients, C1QC^+^ tissue-resident macrophages and CD4^+^ T cells form a spatial niche characterized by significantly reduced intercellular distances and enhanced physical interactions [115]. CD4^+^ T cells located in close proximity to C1QC^+^ macrophages highly express activation markers (CD38, GZMB, TNFα) and also PD-1, indicating a highly activated but potentially exhausted state. In non-responders, however, cancer-associated fibroblasts create a physical barrier that disrupts this spatial connection, preventing the macrophage–T cell interaction and thereby blunting the antitumor immune response [115,116,117]. This discovery illustrates that spatial organization—not merely cell composition—determines immune functionality, and that stromal elements can actively shape immune exclusion by altering tissue architecture.

### 6.3. Spatial Distribution of Metabolic Gradients

The CRC TME is not metabolically uniform; it exhibits steep gradients that profoundly influence immune cell function along a spatial continuum [115]:

Tumor core: Severest hypoxia, highest glycolytic activity, and maximal lactate concentration. Immune cells here are most suppressed—CD8^+^ T cells show profound exhaustion, Treg cells are enriched, and antigen-presenting cells are dysfunctional. The acidic, lactate-rich environment stifles effector functions and reinforces immune checkpoint expression [48,105].

Invasive front: A transitional zone where metabolic gradients attenuate. Here, some immune cells retain partial functionality, and the relative abundance of nutrients (glucose, glutamine) is higher than in the core. This region often exhibits the highest density of infiltrating lymphocytes and is a critical interface for immune–tumor interactions [117].

Distal stroma: Approaching normal tissue, with near-physiological oxygen and nutrient levels. T cells here maintain relatively intact metabolic fitness and effector capacity, although they may be excluded from the tumor core by physical and metabolic barriers.

Induced metabolic bioluminescence imaging (imBI) has confirmed that lactate levels increase progressively from normal mucosa through adenomas and non-metastatic adenocarcinomas to metastatic CRC. Moreover, MCT4 expression correlates positively with lactate concentration, and single biopsies can accurately classify tumors as high- or low-lactate in 83% of cases. These gradients shape the spatial distribution of immune cell phenotypes—different “battlefields” confer different “combat capabilities” on the same cell types. The spatial metabolic gradients and immune cell distribution in the CRC TME are shown in Table 7.

### 6.4. Clinical Implications of Spatial Heterogeneity

#### Clinical Relevance of Spatial Heterogeneity

Biopsy site matters: A single biopsy may not capture the full metabolic landscape of a tumor. If the biopsy is taken from a region with low metabolic stress, biomarkers such as PD-L1 or lactate levels may be underestimated, potentially misclassifying patients for immunotherapy [118].

Differential combination strategies by zone: The spatial heterogeneity suggests that one-size-fits-all interventions are suboptimal. For example, at the invasive front where fibroblasts form physical barriers, targeting fibroblast activation or stromal remodeling may be more effective; at the tumor core, metabolic interventions (e.g., LDHA inhibitors, MCT blockers, or lactate-neutralizing strategies) to relieve immune suppression would be prioritized [105,119].

Predictive biomarker development: Spatial metabolomics can identify metabolic signatures associated with clinical outcomes—for instance, high lactate levels in the tumor core might predict poor response to checkpoint inhibitors. By integrating spatial metabolite profiles with immune cell spatial distribution, we may develop robust predictive algorithms for patient stratification [100,109].

Spatial metabolomics, for all its promise, confronts profound technical and biological limitations that temper its clinical utility [111,113]. The spatial landscape captured by a single biopsy is merely a static snapshot; chemotherapy, radiotherapy, or even PD-1 blockade can radically reshape lactate gradients and immune infiltration within days, rendering baseline “zone-specific” strategies obsolete. Moreover, metabolites such as lactate and ATP have half-lives of seconds to minutes, and routine tissue processing (excision, freezing, sectioning) introduces substantial degradation, so that reported gradients may drastically underestimate in vivo differences. Spatial proximity between C1QC^+^ macrophages and CD4^+^ T cells, though associated with response, does not guarantee functional efficacy—the same neighborhood often harbors potent inhibitory ligands (PD-L1, FasL) and acidic pH that override any beneficial cell–cell crosstalk [120]. Finally, single-site biopsies carry an unacceptable risk of misclassification; to overcome this, multi-region sampling should be integrated with whole-tumor metabolic imaging (e.g., ^18^F-FDG PET/CT or dynamic contrast-enhanced MRI) and serial liquid biopsies for circulating metabolites, thereby avoiding the trap of extrapolating local features to global therapeutic decisions [121].

### 6.5. The “Metabolic Oasis” Concept: Spatial Overlap of Nutrient Availability and Immune Fitness

The identification of C1QC^+^ tissue-resident macrophages and CD4^+^ T cells forming functional niches in immunotherapy responders gains deeper mechanistic meaning when overlaid with the spatial metabolic gradients described earlier [115]. Notably, these immune-rich niches are predominantly located at the invasive front—a region characterized by intermediate oxygenation (pO_2_ ~10–20 mmHg), improved glucose availability (lactate ~5–10 mM), and proximity to functional blood vessels. This location starkly contrasts with the necrotic tumor core, where lactate exceeds 20 mM and pH drops to the lower end of the acidic range (approximately pH 6.0–6.3). We propose that the invasive front constitutes a “metabolic oasis”—a spatially restricted zone where metabolic conditions approach the threshold of T-cell tolerance, thereby permitting the persistence of antigen-experienced CD8^+^ and CD4^+^ T cells and their productive crosstalk with C1QC^+^ macrophages. In non-responders, cancer-associated fibroblasts and dense collagen deposition physically displace this oasis or create diffusion barriers that restrict nutrient influx, effectively converting a potential immune hot spot into a metabolically desertified region. This spatial–metabolic framework has direct therapeutic implications: rather than uniformly targeting lactate throughout the tumor, strategies could be spatially refined to protect or expand the “oasis” (e.g., by using vessel-normalizing agents to improve perfusion at the invasive front) while simultaneously neutralizing the lactate-rich core with MCT4 inhibitors or lactate-oxidizing enzymes. Future spatial metabolomics studies should map not only metabolite concentrations but also the exact coordinates of C1QC^+^ macrophages relative to blood vessels and lactate gradients, to prospectively validate whether “oasis preservation” correlates with progression-free survival.

To translate the ‘metabolic oasis’ concept into clinical practice, several feasible approaches for identification and sampling can be envisioned. Imaging-guided multipoint biopsy—combining preoperative metabolic imaging (e.g., ^18^F-FDG PET/CT or hyperpolarized ^13^C-pyruvate MRI) with intraoperative navigation—could systematically sample the invasive front, tumor core, and distal stroma, enabling spatial mapping of lactate gradients and immune cell distribution. Spatial transcriptomics and metabolomics on these biopsied regions could further characterize the molecular features of the oasis versus the immunosuppressive core. Alternatively, computational modeling integrating imaging data with spatial omics profiles could predict oasis locations non-invasively. While these approaches remain primarily research-oriented at present, ongoing advances in spatial profiling technologies and imaging modalities are rapidly making such spatially refined strategies feasible. Future prospective studies should validate whether preservation or expansion of the ‘metabolic oasis’—for example, through vessel-normalizing agents or localized metabolic interventions—correlates with improved immunotherapy outcomes [115].

## 7. Integrative Framework: Metabolic Competition–Driven Immune Suppression and Therapeutic Prospects

### 7.1. Multi-Level Integration from Molecules to Niches

Metabolites (lactate, lipids, amino acids) act as signaling molecules (GPR81), epigenetic substrates (histone lactylation), and regulators of metabolic checkpoints (IDO, Arg1, LAG-3, TIM-3). These molecular events translate metabolic status into transcriptional and functional changes in both tumor and immune cells [122,123,124].

Different immune cell subsets exhibit distinct metabolic dependencies and sensitivities. CD8^+^ T cells are glucose-dependent and vulnerable to hypoglycaemia [125]; Treg cells thrive on fatty acids and compete effectively under nutrient stress [126]; TAMs adopt context-dependent polarization states influenced by lactate and lipids [127]; MDSCs are regulated by isoform-specific LXR signaling [128]. These differential metabolic profiles determine which immune cells dominate in various TME regions.

Spatial gradients of oxygen, glucose, lactate, and lipids create distinct microenvironments within the same tumor. The physical organization—whether immune cells can access metabolic “hot spots” and whether stromal barriers exclude them—determines the overall immune landscape. The discovery of C1QC^+^ macrophage–CD4^+^ T cell niches illustrates that spatial proximity is a prerequisite for effective immune surveillance [129,130].

**Figure 4:**
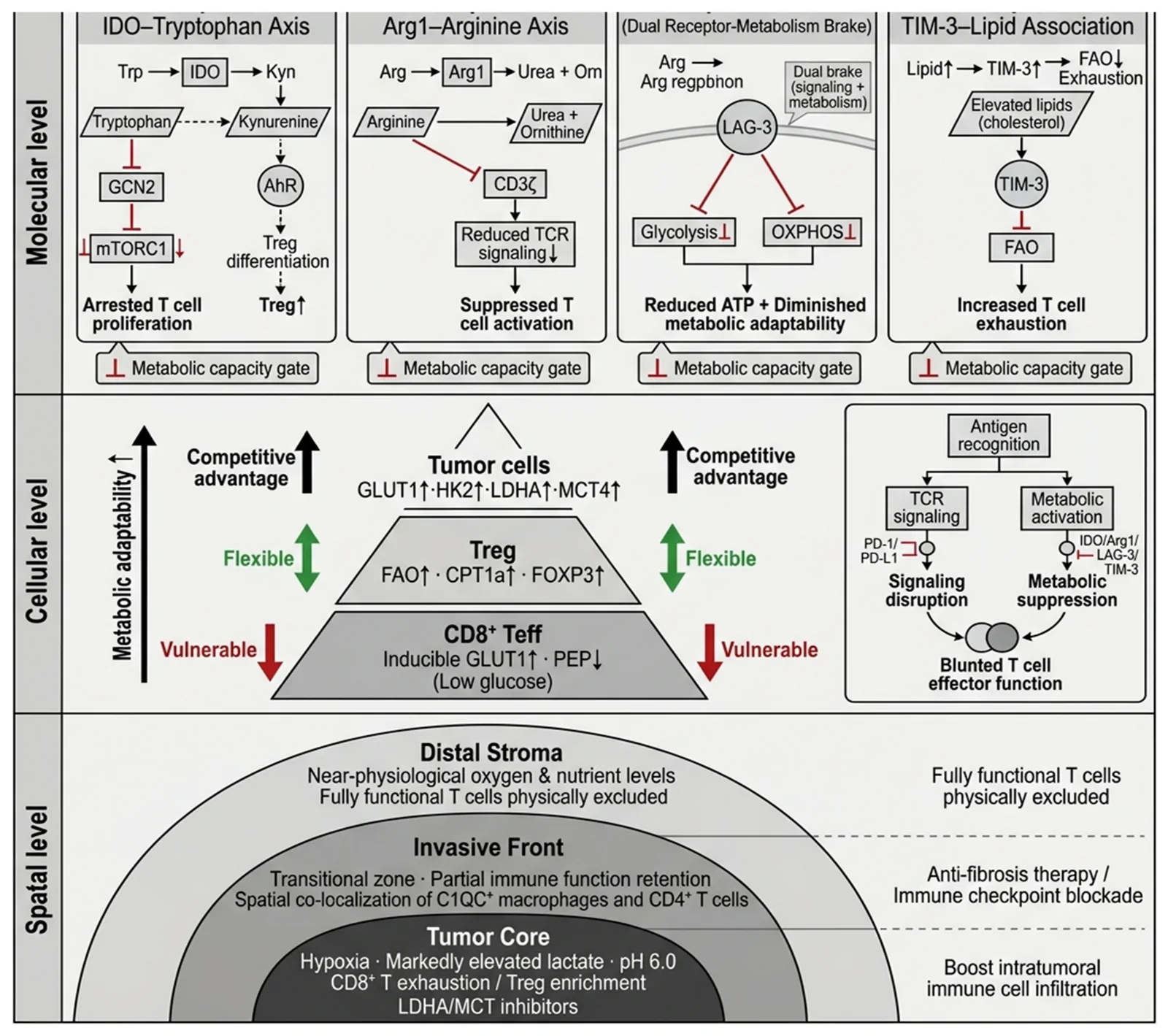
Integrated framework of metabolic–immune regulation and spatial heterogeneity in CRC. Arrows (↑ and ↓) indicate increase and decrease, respectively. Figure 4 was created with Figdraw (www.figdraw.com).

Integrating these three levels is essential for a holistic understanding of how metabolism shapes antitumor immunity and for designing rational combination therapies as summarized in Figure 4.

### 7.2. Unresolved Questions in CRC TME Metabolism Research

#### 7.2.1. Optimization of Combination Regimens

The optimal timing, dosage, and sequence of metabolic interventions combined with immune checkpoint blockade have not been standardized. Should LDHA inhibitors be administered before, concurrently with, or after PD-1 blockade? What are the windows of opportunity?

#### 7.2.2. Relevance of Preclinical Models

Do organoids and mouse models accurately recapitulate the human CRC metabolic TME? Murine tumors often differ in glucose consumption, lactate production, and immune cell composition. Translating preclinical findings to patients remains challenging [131,132].

#### 7.2.3. Causality, Model Limitations, and Translational Hurdles

A critical limitation of the current literature is the difficulty in distinguishing causal relationships from correlative associations. The majority of evidence presented in this review—including the links between lactate accumulation, histone lactylation, and immune suppression—derives from cell line studies or murine models, and their direct relevance to human CRC remains to be rigorously established. While mouse models and organoid systems have provided valuable mechanistic insights, they often fail to recapitulate the full complexity of the human TME, including the three-dimensional stromal architecture, the diversity of immune cell infiltrates, and the dynamic metabolite fluxes that characterize patient tumors [133,134].

Translating lactate-targeting strategies to the clinic faces several substantive hurdles. First, compensatory pathways represent a major challenge. LDHA inhibition may be counterbalanced by LDHA-independent lactate production or compensatory upregulation of MCT1 [133,134,135]. Similarly, MCT4 blockade may be offset by MCT1 upregulation on both tumor and immune cells [135]. Second, safety concerns are non-trivial: MCT1 is essential for T-cell activation and proliferation, and its systemic inhibition could inadvertently suppress antitumor immunity [136,137]. LDHA, while an attractive target, is ubiquitously expressed in normal tissues, raising concerns about off-target toxicities. Histone deacetylase inhibitors (e.g., panobinostat), despite showing promising effects in reducing H3K18 lactylation and PD-L1 expression in preclinical CRC models, are associated with significant dose-limiting toxicities and off-target effects on other epigenetic modifications [135,138].

Third, the lack of validated predictive biomarkers represents a major bottleneck. While tumor lactate levels, MCT4 expression, and H3K18la status have been proposed as potential biomarkers, their utility in patient selection remains unproven [139]. Furthermore, single-biopsy approaches are insufficient to capture the intratumoral heterogeneity of lactate gradients, potentially misclassifying patients [138].

Fourth, the causal hierarchy of metabolic alterations in the TME remains unresolved. It is plausible that many observed metabolic changes are secondary consequences of an already immunosuppressive microenvironment rather than primary drivers of immune evasion. This ambiguity has direct therapeutic implications: if lactate accumulation is primarily a consequence rather than a cause of immune suppression, then targeting lactate may yield limited clinical benefit unless combined with interventions that address the underlying immune dysfunction. Prospective “window-of-opportunity” trials—in which patients receive short-term metabolic inhibitors prior to surgery, with paired biopsies collected before and after treatment—are urgently needed to directly track the temporal relationship between metabolite shifts and T-cell functional recovery [139].

Together, these considerations underscore the need for a cautious and nuanced approach to clinical translation. While the preclinical promise of LDHA/MCT4 inhibition and lactylation modulation is substantial, their successful integration into CRC immunotherapy will require careful patient selection, rational combination strategies, and a clear-eyed assessment of both the therapeutic potential and the inherent limitations of targeting a metabolically redundant and physiologically essential pathway [34].

Are metabolic alterations drivers of immune suppression, or are they merely consequences of an already immunosuppressive TME? Disentangling cause from effect is crucial for prioritizing therapeutic targets [10,140,141,142].

### 7.3. Future Research Directions

#### 7.3.1. Multi-Omics Integration

Combining single-cell metabolomics, spatial metabolomics, and proteomics will generate comprehensive “metabolic-immune atlases” of CRC. Such atlases will map metabolite fluxes, immune cell states, and their spatial relationships at the single-cell resolution, enabling identification of actionable vulnerabilities [143,144].

#### 7.3.2. Biomarker-Driven Clinical Trial Design

Metabolic biomarkers—such as serum LDH, tumor lactate levels by imBI, or MCT4 expression—should be used to select patients most likely to benefit from metabolic–immune combinations. The RECOURSE trial has already shown that low LDH correlates with better overall survival in refractory mCRC patients receiving trifluridine/tipiracil, validating the prognostic power of metabolic markers.

#### 7.3.3. Targeting “Irreversible Checkpoints”

Once T cells enter terminal exhaustion or a stable Treg-like fate, their plasticity diminishes dramatically. Early intervention to protect T cells from metabolic “domestication” may be more effective than trying to reverse established dysfunction. Thus, metabolic therapies should be integrated upfront, not as salvage regimens.

#### 7.3.4. Paradigm Shift

Moving from “activating T cells” to “protecting T cells from metabolic hijacking”, rather than solely attempting to boost T-cell activity, we should shield them from tumor-driven nutrient competition and metabolic poisoning (by lactate, lipids, and acid). This entails not only inhibiting tumor glycolysis but also normalizing the TME’s metabolite landscape—for example, by neutralizing lactate, reducing lipid overload, or supplying alternative fuels to immune cells (Table 8).

At the integrative level, we must candidly acknowledge that nearly all the evidence discussed is correlational, not causal, in human disease. The field urgently needs prospective “metabolic intervention window” trials—short-term preoperative administration of LDHA or MCT inhibitors paired with paired biopsies before and after treatment to directly track the temporal relationship between metabolite shifts and T-cell functional recovery. Patient-derived tumor explant cultures that preserve autologous immune cells and stromal architecture could serve as an ex vivo platform to test individualized metabolic perturbations coupled with single-cell functional readouts. Above all, we should resist metabolic reductionism: nutrient competition is only one node in an intricate network of genetic mutations, microbiota, and neuro-immune signals. Metabolic therapies are necessary but rarely sufficient; they must be rationally sequenced with classical immune checkpoint blockers, epigenetic modulators, or tumor vaccines, and continuously adjusted based on dynamic biomarkers. Only by embracing this complexity—and by discriminating causality from association—can we transform the current metabolic–immune atlas into a practical roadmap for overcoming immunotherapy resistance in colorectal cancer.

## Figures and Tables

**Table 1:** Metabolic demands of immune subsets.

Feature	Tumor Cell	CD8^+^ Effector T Cell	Treg Cell
Primary metabolic mode	Aerobic glycolysis (Warburg effect)	Resting: OXPHOS; Activated: aerobic glycolysis	FAO + OXPHOS (facultative glycolysis)
Glucose dependence	high, but plastic	high, no alternative	moderate, fuel-switching
Metabolic flexibility	can utilize glutamine/fatty acids	low, single-fuel dependent	moderate, FAO as alternative
Key transporters/enzymes	GLUT1, HK2, LDHA, MCT4	GLUT1 (inducible), PEP (glycolytic intermediate)	AMPK, PPARγ, CPT1a
Adaptation to glucose limitation	Low K_m_, high V_max_ uptake; switches to alternative substrates	No effective adaptation → functional failure	FAO/FAS support energy supply → FOXP3 stabilization
Key reaction	C_6_H_12_O_6_ + 2ADP + 2Pi → 2C_3_H_6_O_3_ + 2ATP	PEP + ADP → Pyruvate + ATP	Palmitoyl-CoA + 7FAD + 7NAD^+^ +7CoA → 8Acetyl-CoA + 7FADH_2_ + 7NADH + 7H^+^
Competitive outcome	Absolute advantage	Disadvantaged, vulnerable to deprivation	Relative advantage (under low-glucose, lipid-rich conditions)

**Table 2:** Comparison of MSS and MSI-H Colorectal Cancer [1,2,13].

Feature	MSS CRC	MSI-H CRC
Prevalence	~85%	~15%
Tumor mutational burden	Low	High
Common driver mutations	KRAS (30–50%), BRAF (5–15%)	BRAF V600E, APC
Consensus Molecular Subtype	CMS2/3/4	CMS1
Immune phenotype	“Cold”/immune-excluded	“Hot”/immune-infiltrated
Key immune infiltrates	Tregs, MDSCs, M2-TAMs, CAFs	CD8^+^ T cells, NK cells
ICI responsiveness	Poor/refractory	Favorable
Glycolytic activity	High (KRAS/BRAF-driven)	Lower
Lactate accumulation	High	Lower
Lipid metabolic features	Enhanced FAO, cholesterol reprogramming	Reduced cholesterol homeostasis

**Table 3:** Triple Immunosuppressive Mechanisms of Lactate in CRC.

Mechanistic Dimension	Core Mediator/Receptor	Primary Target Cells	Key Molecular Events	Immunosuppressive Consequences	Intervention Strategies
Signaling	GPR81 (HCAR1)	CRC cells, TAMs, MDSCs	Inhibits cAMP/PKA pathway; induces CCL2/CCL7 secretion	Recruits PMN-MDSCs; suppresses T-cell proliferation and cytokine production	Reserpine (HCAR1 inhibitor) + anti-PD-1 (tumor shrinkage in models)
Epigenetic	Histone H3K18 lactylation	CRC cells, CD8^+^ T cells, TAMs	PD-L1 promoter lactylation (expression upregulated); silencesIFN-γ/GZMB; upregulates METTL3–JAK1–STAT3 axis	Induces T-cell exhaustion; promotes M2 TAM polarization; enhances autophagy and bevacizumab resistance	HDAC inhibitors (panobinostat), antifungal sulconazole
Acidification	Lactate accumulation (pH 6.0–6.5)	NK cells, CD8^+^ T cells, tumor cells	Inhibits glycolytic enzymes (e.g., phosphofructokinase); disrupts TCR signaling	Diminishes NK cytotoxicity; upregulates PD-L1; drives T-cell dysfunction	Lactate-neutralizing antibodies, nanoparticle-encapsulated MCT4 siRNA

**Table 4:** Lipid Metabolism Differentially Regulates Immune Cell Subsets in CRC.

Immune Subset	Microenvironmental Lipid Status	Major Metabolic Alteration	Key Molecules/Pathways	Functional Outcome
CD8^+^ T cells	Excessive cholesterol esters (CE)	ACAT1-mediated esterification traps free cholesterol in lipid droplets; plasma membrane cholesterol “starvation”	ACAT1, ER stress, mitochondrial dysfunction	Impaired TCR signaling; reduced cytokine secretion; lipotoxicity-driven exhaustion
Treg cells	Fatty acid-rich	Upregulated FAO and FAS; reliance on OXPHOS	AMPK ↑, PPARγ ↑, CPT1a ↑; FOXP3 stability enhanced	Increased metabolic fitness; survival advantage under low-glucose, high-lipid conditions
TAMs	Cholesterol/fatty acids (highly context-dependent)	Cholesterol uptake; CYP27A1 generates 25-hydroxycholesterol	STING pathway (aberrant activation), LXR signaling	Promotes M2 polarization and CD8^+^ T-cell exhaustion; promotes metastasis and immunotherapy resistance in CRC [79]
MDSCs	Oxysterols	Differential LXRα vs. LXRβ activation	LXRα → pro-suppressive; LXRβ → pro-antitumor	Opposing functions within the same pathway

Note: ↑ indicates upregulation; → denotes “leads to” or “results in.” These symbols are used for qualitative representation only.

**Table 5:** IMMETCOLS Metabolic Subtypes in MSS CRC.

Subtype	Prevalence	Core Metabolic Feature	Therapy Resistance
IMC1	~35%	Glycolysis + TGF-β signaling (inflammatory fibroblasts)	Chemotherapy
IMC2	—	OXPHOS + glutamine metabolism (antioxidant defense)	Cytotoxic agents, anti-EGFR
IMC3	—	Lactate-fueled respiration + PPP activation	Targeted therapy (anti-BRAF/KRAS)

**Table 6:** Key Metabolic Checkpoints in the CRC Microenvironment.

Metabolic Checkpoint	Key Enzyme/Receptor	Target Substrate	Direct T-Cell Suppression Mechanism	Synergy with Classical Checkpoints
IDO–tryptophan axis	IDO1 (indoleamine 2,3-dioxygenase)	Tryptophan → kynurenine	Tryptophan depletion → GCN2 → mTORC1 inhibition; kynurenine activates AhR → promotes Treg differentiation	Feedback loop with PD-1/PD-L1 (IFN-γ induces IDO)
Arg1–arginine axis	Arg1 (arginase-1, high on MDSCs)	L-arginine → urea + ornithine	Arginine deprivation → reduced CD3ζ chain → impaired TCR signaling; suppresses mTORC1	Arginine starvation upregulates tumor PD-L1, reinforcing PD-1 axis
LAG-3–metabolism axis	LAG-3 (lymphocyte activation gene-3)	MHC-II binding	Competes with CD4 for MHC-II binding with higher affinity, blocking CD4^+^ T-cell helper signals; intracellular KIEELE motif transduces inhibitory signals, suppressing proliferation and cytokine production; also inhibits glycolysis and mitochondrial OXPHOS, reducing metabolic fitness	LAG-3 and PD-1 are co-expressed on exhausted T cells; dual blockade with anti-LAG-3 (e.g., relatlimab) and anti-PD-1 (nivolumab) shows synergistic restoration of T-cell function
TIM-3–lipid axis	TIM-3 (T-cell immunoglobulin and mucin-domain containing-3)	Ligands (galectin-9, etc.)	Ligands (galectin-9, HMGB1, CEACAM1, phosphatidylserine) engage TIM-3 and induce inhibitory signaling via Tyr256/263 phosphorylation in the cytoplasmic tail, suppressing T-cell proliferation and effector function; cholesterol-rich microenvironment upregulates TIM-3 expression and interferes with FAO and mitochondrial respiration, driving exhaustion	TIM-3 is frequently co-expressed with PD-1 on exhausted T cells; combined TIM-3/PD-1 blockade synergistically restores T-cell effector function in preclinical models

**Table 7:** Spatial Metabolic Gradients and Immune Cell Distribution in the CRC TME.

Spatial Zone	Oxygenation	Glucose/Lactate Levels	Dominant Metabolic Features	Immune Cell Phenotype and Function
Tumor core	Severe hypoxia	Glucose very low; lactate > 20 mM (necrotic); pH 6.0–6.5	Highest glycolysis; LDHA/MCT4 high; maximal H3K18 lactylation	CD8^+^ T deeply exhausted (PD-1^+^TIM-3^+^); Treg enriched; DC dysfunction
Invasive front	Moderate hypoxia (gradient)	Nutrients relatively improved; lactate ~5–10 mM	Intermediate metabolic activity; MCT4 expression correlated with core	Highest density of CD8^+^/CD4^+^ T cells; C1QC^+^ macrophage–CD4^+^ T cell functional niches; fibroblast barriers
Distal stroma	Near-physiological	Near normal (lactate baseline levels)	OXPHOS dominant	T cells metabolically intact but physically excluded from tumor core

Note: Interstitial fluid lactate concentrations (mM) are presented in this table, as these directly reflect the biochemical milieu of immune cells. For reference, these interstitial levels (e.g., >20 mM in the core) correspond to tissue homogenate values of approximately 30–40 µmol/g wet weight, depending on the extracellular volume fraction (φ ≈ 0.2–0.3).

**Table 8:** Core Clinical Translation Challenges and Future Strategies for Metabolic Interventions.

Intervention Target	Core Clinical/Translational Hurdles
Lactate metabolism (LDHA/MCT)	Biphasic, concentration-dependent effects (1–5 mM promotes memory T cells; >15 mM suppresses); ② MCT1 indispensable for normal T-cell activation
Cholesterol/lipid metabolism (ACAT1/LXR)	① ACAT1 maintains adrenal and intestinal homeostasis; chronic blockade risks steroid deficiency and colitis; ② LXRα/β overlap in hepatic reverse cholesterol transport
Metabolic checkpoints (IDO/Arg1)	Physiological IDO protects placenta, CNS, and gut mucosa; ② TDO compensation and direct tumor uptake of kynurenine bypass IDO blockade
Spatial sampling and biomarkers	Metabolites have very short half-lives (seconds–minutes); routine processing grossly underestimates in vivo levels; ② Single biopsy misses intratumoral heterogeneity (misclassification risk ~17%)
Causality and model reliability	Most evidence is correlational, not causal; ② Mouse models differ from human CRC in glucose consumption and immune composition

## Data Availability

No new data were created or analyzed in this study. Data sharing is not applicable to this article.

## Abbreviations

The following abbreviations are used in this manuscript:

ACAT1	Acyl-CoA Cholesterol Acyltransferase 1
AhR	Aryl Hydrocarbon Receptor
Arg1	Arginase 1
C-6-S	Chondroitin-6-Sulfate
CAF	Cancer-Associated Fibroblast
CRC	Colorectal Cancer
CTLA-4	Cytotoxic T-Lymphocyte-Associated Protein 4
FAO	Fatty Acid Oxidation
FAS	Fatty Acid Synthesis
FASN	Fatty Acid Synthase
IFN-γ	Interferon-gamma
GZMB	Granzyme B
GLUT1	Glucose Transporter 1
GPR81	G-Protein-Coupled Receptor 81 (also known as HCAR1)
HCAR1	Hydroxycarboxylic Acid Receptor 1 (same as GPR81)
HDAC	Histone Deacetylase
HIF-1α	Hypoxia-Inducible Factor 1-alpha
HK2	Hexokinase 2
ICIs	Immune Checkpoint Inhibitors
IDO	Indoleamine 2,3-Dioxygenase
IMC	Imaging Mass Cytometry
ISCU	Iron-Sulfur Cluster Assembly Enzyme
LAG-3	Lymphocyte Activation Gene-3
LDHA	Lactate Dehydrogenase A
LXR	Liver X Receptor
mCRC	Metastatic Colorectal Cancer
MCT4	Monocarboxylate Transporter 4
MDSCs	Myeloid-Derived Suppressor Cells
MSI-H	Microsatellite Instability-High
MSS	Microsatellite-Stable
mTORC1	Mechanistic Target of Rapamycin Complex 1
NK	Natural Killer (cells)
OXPHOS	Oxidative Phosphorylation
PD-1	Programmed Cell Death Protein 1
PD-L1	Programmed Death-Ligand 1
PEP	Phosphoenolpyruvate
PMN-MDSCs	Polymorphonuclear Myeloid-Derived Suppressor Cells
SERCA	Sarco/Endoplasmic Reticulum Ca^2+^-ATPase
SREBF1	Sterol Regulatory Element-Binding Transcription Factor 1
TAMs	Tumor-Associated Macrophages
TCA	Tricarboxylic Acid
TCR	T-Cell Receptor
TIM-3	T-Cell Immunoglobulin and Mucin-Domain Containing-3
TME	Tumor Microenvironment
Tregs	Regulatory T Cells
